# Communicative and Discursive Perspectives on the Medication Experience

**DOI:** 10.3390/pharmacy9010042

**Published:** 2021-02-17

**Authors:** Lewis H. Glinert

**Affiliations:** Department of Middle Eastern Studies and Linguistics, Dartmouth College, Hanover, NH 03755, USA; Lewis.Glinert@Dartmouth.edu

**Keywords:** language, discourse, communication, medication, health literacy, labelling, advertising, internet

## Abstract

Taking the ‘medication experience’ in the broad sense of what individuals hear and say about their medication, as well as how they experience it, this paper explores diverse research on medication information available to patients and their modes and capacities for interaction, including personal circles, doctors and pharmacists, labeling and promotion, websites, and the patient’s own inner conversations and self-expression. The goal is to illustrate, for nonspecialists in communication, how the actors, messages, mediums, genres, and contextual factors within a standard ethnographic and social semiotic model of discourse and communication are operating, not always effectively or beneficially, to mediate or construct a patient’s medication experience. We also suggest how disparate insights can be integrated through such a model and might generate new research questions.

## 1. Introduction: Key Concepts

### 1.1. Communication and Health Communication

This paper seeks to illustrate, for the nonspecialist in communication, how the medication experience is expressed through communication and discourse. Discourse is the process of conveying meaning by language, including how it is intended and how it is taken. Discourse, in turn, forms part of communication, embracing the multitude of factors that affect the discourse process, such as role, goals, and setting. Research in the discourse of medication and ‘meds talk’ is significant but not copious; two recent handbooks of health communication and medical anthropology [1,2] did not think the subject worthy of a chapter. After presenting a minimal model of the participants in health communication and a general model of communications, we examine a broad informal sample of studies pertaining to the discourse of the medication experience, positioning them within these models. This in turn will permit us to suggest lines for further research.

**Figure 1:**
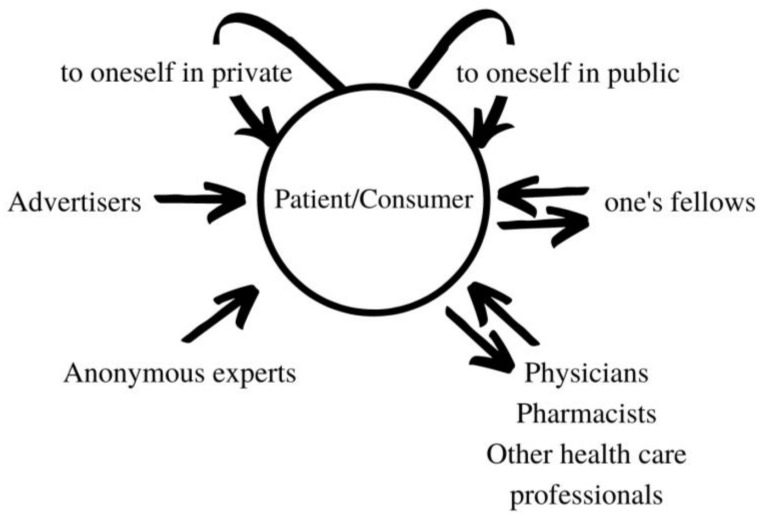
Parties to health messages to the patient/consumer.

The shift to patient-centered values in recent decades has coincided with a ‘turn to language’ in medical research. The study of health communication is now widely conceived as a master key to patient-centered health care delivery. For many, the prime focus, quoting the foreword to a recent handbook of health communication [1], is ‘education and research in the field of doctor-patient relationships and communication’. However, this, we shall argue, is a serious misconception. Health communication for the patient and the consumer, and in particular communication about the medication experience, involves much more than the doctor and patient. A minimal model of the parties to health messages must include (Figure 1).

But what are these messages intended to mean and achieve? And what factors play a part, at the sender’s and the recipient’s end? This is the concern of a general model of communication. Early models of communication, portraying ‘packets’ of ‘information’ being encoded by a sender and travelling via a medium to a recipient for decoding, were swiftly superseded by far more complex models, involving many more factors and problematizing the very function of communication [3,4,5].

**Figure 2:**
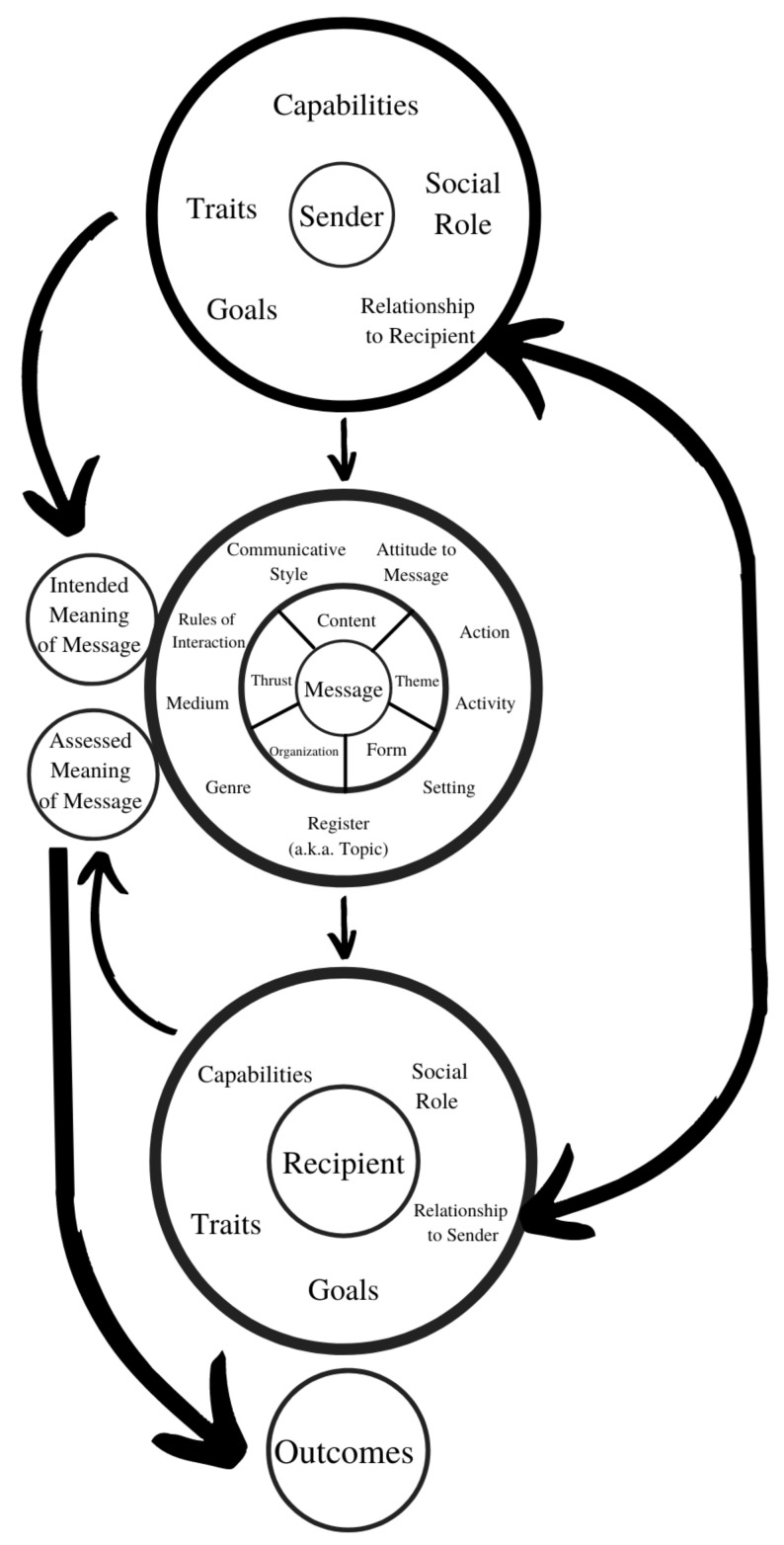
Model of general communication.

One powerful and influential model developed by linguists and discourse-oriented social scientists starting in the 1960s is the ethnographic-sociolinguistic model [3,6,7,8] (Figure 2). 

The ethnographer of language asks what is ‘being done’ by what is said and by all the contributory elements of the message proper (the inner circle around ‘message’) and the situation (the outer circle), while the sociolinguist is concerned with the social categories that affect and reflect discourse (in the circle around ‘sender’ and ‘recipient’). 

An altogether broader view of the health communication process, widely used by social psychologists [9], would include the sender’s and recipient’s values, beliefs, and cognitive traits. For example, according to protection motivation theory [10], a sense of vulnerability, of severe threat, of the efficacity of the health action, and of one’s ability to adopt it should improve response to health-related messages; the transtheoretical model [11,12] identifies decision-making stages, such as precontemplation, contemplation, and action, as a precursor to changing one’s health behavior; and the advisor for risk communication (ARC) model developed by Keller and Lehmann [9] identifies 10 variables predicting healthy intentions—four audience characteristics (age, gender, race, and regulatory focus, e.g., growth vs. security) and six message tactics (health, gain/loss, physical and social consequences, impact on self vs. others, emotion, vividness). Such factors would appear to lie largely beyond the scope of our model and its application to medication communication, except to the extent that anthropologists and discourse analysts find them in actual ‘medication talk’ (see Section 1.3)

Note that in Figure 2, the sender forms an intended meaning and a suitable message-with-context to convey it, and the recipient duly constructs an interpretation. This and the outcome of the communication may not match the sender’s intent and, indeed, may draw a host of inferences not intended by the sender.

The factors in Figure 2 are enumerated with examples in Table 1 (the ordering not having any strict significance). Reference to these factors will be made by # throughout the paper.

These factors can work as independent variables, but there can be substantial interaction. For example, the tone (#8), genre (#9), and content and form of the message (#6) often encode or, conversely, affect roles and relationships (#2). Thus, a casual tone can reflect the doctor–patient relationship, but it can also be employed to alter it.

We shall also be noting a further related dimension of the meaning of medications for patients: the semiotic dimension, i.e., meaning-making in its broadest sense, be it within language or within some other symbolic system [13,14], such as images of foods, clothes, or medicinal pills. Here, contrast and associativity are major factors in meaning. A sub-field of social semiotics has been developed to study how meaning-making operates in personal interaction [15,16]. 

For better or for worse, the study of communication in the past 40–50 years has become a vast enterprise, something of an intellectual and institutional mêlée, due in part to the variety of disciplines that have declared an interest in language and communication, ranging from biology to media studies. The study of communication has been described as a combination of fields, based on a mix of scholarly-disciplinary traditions, rather than a discipline with an integrated conceptual rationale [17]. Thus, the Reader’s Guide to a recent encyclopedia of communication theory [18] lists close to 1000 sub-theories and concepts, from advertising theories and argumentation theories to symbolic interactionism and values theory. 

Health communication is no less varied. As one theoretician has observed [19]:

Health communication scholars apply a wide range of different theories, models, and research methods from these different areas of communication inquiry to examine health communication phenomena. They also adopt theories and methods that derive from many other related disciplines, such as psychology, sociology, anthropology, public health, medicine, nursing, health education, epidemiology, and social work.

This paper seeks to narrow the focus and sharpen the conceptual perspective in terms of the communications model outlined above.

The ‘medication experience’ has been conceptualized [20] as embracing, at its broadest, objective medication history, current medications, and associated conditions, and how the patient perceives them. A narrower definition, more apt to our mind, is ‘an individual’s subjective experience of taking a medication in his daily life’ [21], meaning the patient’s personal approach to the use of these medications, including wants and expectations, concerns, grasp of the drug therapy, and medication taking behavior. Patently, the term ‘experience’ is problematic, variously covering (both as ‘an experience’ and ‘to experience’) different parts of the subjective–objective spectrum. Associated, albeit distinct, issues are whether the experience is one’s own or others’, and real or imagined. We shall therefore broaden our scope to include the discourse of the consumer’s a priori (anticipatory) as well as a posteriori experience of medication. What the patient says and what the patient hears is arguably part of a loop, and yesterday’s anticipatory incoming messages will often become part of an ongoing medication conversation.

### 1.2. Order of Presentation

The organization of this paper proceeds from the small scale, names and words (Section 2 and Section 3), through modulations of meaning by suggestivity and emphasis (Section 4 and Section 5), to ‘default’ communication: messages in context. Here, we proceed from interactive communication, mediated by factors from the broad (Section 6: participants, activity, setting) to the narrower (Section 7 and Section 8: traits, capabilities, sociocultural effects), to ‘one-way’ communication with the self (Section 9) and one-way quasi-anonymous incoming communication by way of written patient information (Section 10 and Section 11), and finally—sui generis and uncharted seas—the Internet and social media.

### 1.3. Discourse’ and ‘Meaning’

Two particular foci of this paper are *discourse* and *meaning*, both of them critical drivers in the machinery of communication. These terms have been deployed in diverse, even conflicting ways. We shall use ‘discourse’ (adjective: discursive) in the widely established sense of the process by which a message is constructed and shared between real or imagined parties [22,23,24]. The study of discourse has had to dispel some common misconceptions. Often, a message is primarily intended (and taken up) to reflect or affect a relationship and not to convey information per se. What is said is by no means ‘spelled out’ in a text; it will typically require the recipient to engage in rapid, usually subconscious inferencing. At times, a message may not even have an intrinsic meaning [24]. Indeed, what is said is sometimes strategically intended to be indeterminate; the interacting parties will often need to negotiate what it means, or even choose not to agree on what the meaning is [22]. It should thus be evident that much of what happens in discourse is a far cry from the simple notion of a package of information that gets encoded and decoded. This theory of ‘discourse’ also seeks to capture a host of textual signals that contribute to the interactive process of producing meaning [23], and looks beyond the text proper to verbal context, formatting, images, body language, and so on. Seemingly simple discourse can involve elaborate orchestration.

Within the realm of medicine, the workings of discourse have attracted substantial attention: for example, how medical texts operate institutionally and interpersonally [25] and how doctors interact with patients [26]. Medical discourse is thus a major element in medical communication (note that the term ‘discourse’ is also used of the entire body of knowledge in a field, ‘discourse with a capital D’ as it is sometimes dubbed).

The word *meaning* spans an array of phenomena, all of them central to the content (factor #6 above) of communication but in quite different ways. The fact that they are all referred to by the word ‘meaning’ in English does not in itself mean that they are intrinsically related. They are listed below with examples. Speakers use them freely without usually being able to classify or define them:

1. The denotation: what a word or text means in and of itself, e.g., ‘purple pill’: pill of purple color.
2. The connotation: what it means by association, especially through context, e.g., ‘purple’ can connote nobility, wealth; ‘purple pill’ can have contemporary cultural connotations.
3. The pragmatic meaning: what you are ‘getting at’ by these words in this instance, e.g., ‘Help prevent clot formation’ in a drug ad is meant to justify the plug for the drug.
4. The perlocutionary meaning: what you mean to accomplish by the words, e.g., ‘Help prevent clot formation’ etc.’ in a drug ad aims, ostensibly, to get you to ask your doctor about the drug.
5. The semiotic meaning: what the text or object or action means as part of a system, e.g., purple pills in contrast with pills of other colors (as conceived by consumers, regulators, manufacturers).
6. The significance: what a text or an experience ‘means to me’, in terms of beliefs, values, and other impact.

Words and sentences have meaning even when they are not being actively used to communicate. This is part of one’s communicative competence. In much the same way, one’s experiences can have meaning or ‘significance’, which may or may not be communicated to others.

The ‘meaning’ or significance of the patient’s medication experience resides to some degree in subjective and affective responses, how people think or feel or act, but also in words and objects: at a discursive level and at a ‘semiotic’ level, which can involve but can also transcend discourse [13,14]. Semiotic meaning of a medication can be sought, for example, in the way it looks—say, the shape and color of pills—or in what people say about it.

The meaning of what people ‘say’ about something is much more than the semantics of their words and sentences. A message and its parts are likely to have a ‘point’ or ‘thrust’, and certain words and themes will be salient or backgrounded. Anthropology and cultural analysis may mine for ‘key’ words or salient themes [27]. Thus, Conrad [28], in a pioneering study of perceptions of medication, connected non-adherence with salient themes of ‘not to be dependent’, self-regulation, and ‘I don’t want to be classified’. Although patient and physician may ostensibly ‘speak the same language’ in both the literal and metaphorical sense of the phrase, there may be a gulf between them in understanding what we above termed the ‘pragmatic’ meaning of what the patient says, i.e., what the patient is ‘getting at’: Their physician may construe their actions as ‘noncompliant’, and the patients may attribute to their actions (and the medications themselves) a quite different meaning. Or, more likely, there is an interaction of sorts going on, part of what Halliday [15] terms a social semiotic but constantly morphing as the interaction progresses. By contrast, adherence to a medicine has been found expressed in terms of fear (the invisible disease), self-preservation (‘taking care of oneself’), and variations on a theme of trust: trust in the drug and trust in one’s physician [29].

However, it is possible to look at the relation between meaning and action in reverse: The patient’s actions or talk or narration themselves have the capacity to generate meaning for the patient [30,31]. The world is experienced in the act of languaging it [32,33].

Ultimately, the patient may come to be ‘defined’ by the medication [34]. Or, again [35], patients seemingly powerless to act may identify successive phases of their medication experience, from the initial encounter (e.g. ‘I’m ageing’, ‘I don’t need to take it’), followed by all the bodily effects and the unremitting regimen, to finally mastering the medication and finding it meaningful.

Whatever words or themes a patient may use, their meaning may be partly dependent on a relativistic dimension, the ‘place’ carved out for them in a system of differing words or themes. This aspect of language has been famously likened to playing a game of chess, in which what counts is the function and relationship of the pieces, not their substance [36]. As such, patient perceptions of medicines function much like other social or cultural systems, such as narratives, images, cities, foods, ceremonies, and kinships [37,38,39,40,41,42]. Thus, one drug may derive ‘meaning’ by comparison with another, or compared as a type with other types, over-the-counter vs. prescribed, brand name vs. generic, biomedical vs. complementary, manufactured vs. home remedy. Some people speak of over-the-counter medicines as being unable to cause harm [43] or dismiss an everyday drug for minor ailments, such as paracetamol or an antacid, as ‘less of a medicine’ or a non-medicine [44,45], a belief that may be bolstered by a sense that prescription drugs by comparison are high risk. Medicalization of a condition, such as attention-deficit/hyperactivity disorder, may also impact how a medication is spoken of and dealt with [46].

## 2. General Descriptors for Medications

Let us begin at a very small scale, the individual word, and ostensibly with a banal matter: the choice of words to denote ‘medication’ (or a particular kind of medication), such as ‘medicines’, ‘medications’, ‘meds’, ‘drug’, ‘pill’, ‘tablet’, and ‘remedy’. Are these just trivial variants? Connotation, in general, is not so much a property of words in isolation (as in a dictionary) as of words in communication. These terms appear to involve several of the communication factors listed earlier, such as the sender, receiver, and realm of discourse. Other factors that might be included are culture and demographics. Additionally, we need to distinguish the denotations and connotations (two of the types of meanings described above), while also being on the look-out for shifts in meaning due to the ‘jostling’ between words just described. This area of medication discourse could potentially help predict attitudes and behaviors regarding medications, but it appears to be relatively unexplored.

Consider ‘remedy’ and ‘pill’: Impressionistically, allopathic medications are variously called *drugs*, *medicines*, *medications*, *meds*, *pills*, as against homeopathic *remedies* (the general term employed by homeopaths). However, do non-homeopathic professionals also call them ‘remedies’? And there are home *remedies*. Who uses these terms to whom, how, and when? Do the non-medical connotations of ‘home remedy’ affect the connotations of a homeopathic ‘remedy’? Was there some verbal magic in AstraZeneca’s top-grossing ‘purple pill’ campaign for Nexium? And what descriptors are used for OTC substances [47], vitamins, and herbs?

Further questions could be asked: Are trends toward medicalization [48,49,50] affecting terminology? Conversely, could terminology be a driver of medicalization? (and what are the regulatory implications?) Are expert or lay words like ‘disease’, ‘illness’, ‘ailment’, and ‘complaint’ and for specifics like ‘sleeplessness’ and ‘sleep disorder’ associated with different terms for medications and taking medications, perhaps encoding different degrees of medicalization [51] or pharmaceuticalization. Thus, is someone who complains of ‘poor sleep’ rather than ‘sleep disorder’ or ‘insomnia’ [52] liable to speak of ‘taking *something* for it’ rather than ‘taking a pill’? (of course, the doctor’s own diagnosis may affect the patient’s own thinking). Does saying ‘I’m on x’ betoken a particular kind of medicine, speaker and addressee, attitude to the medicine, and so on? The data may be complex, both quantitatively and qualitatively. Social and cultural factors may be operating. Indeed, there may be no simple pattern. Linguists raised on structuralist or positivist paradigms (dominant until the late 20th century) of clear and relatively consistent linguistic patterning will seek parallels between such sets of terms, but even in the case of broad patterning, a finer examination may reveal variability or instability, reflecting social and personal circumstances, projection of status, and negotiation of power as a conversation progresses [53].

## 3. Do Drug Names Carry Meaning?

Connotation and suggestivity are more in evidence when we turn from common nouns to names. Whether names in general carry meaning depends on the kind of name and on the culture, and on what one means by ‘meaning’. So, too, with the meaning to patients of the names of medicines. Like any other words, common and proper nouns for medicinal substances may sometimes carry cultural or personal connotations as well as denoting a substance. Viagra, for example. Such connotations can be the result of any number of associations. By contrast, chemical drugs are given chemical names and generic names designed to have no denotations or connotations. Brand names, however, are specifically chosen for their *connotational potential*—not their commonplace linguistic connotations but their evocative or aesthetic power or peculiarity or other memorable qualities [54,55]. 

However, beyond these ‘ordinary’ connotations to which users are likely to be attuned, there are less conscious suggestivities. Although medical brands are prohibited by the FDA from suggesting efficacy on an overt linguistic plane [56,57], it is possible for branders to utilize (by design or by instinct) the suggestivity of sound symbolism, which has been empirically demonstrated for particular languages or cultures [58,59] and is familiar to many Americans from, e.g., the use of the ‘streamlined’ sound (and shape) of ‘z’ in auto and high-tech brand names [60]. Thus, one study of commonly used chemotherapy names has found a significantly above-normal incidence of ‘p, t, k’ (unvoiced stops) as against ‘b, d, g’ (voiced stops) [61], often associated in the general data on sound symbolism with speed and lightness, suggesting that the names are signaling to chemo patients that such drugs work swiftly and lightly. The FDA has never sought to apply its regulations on this plane. Further research is called for in this area, with the potential for prompting regulatory attention.

More generally, the attraction and even loyalty and closeness sometimes felt to a drug [29] may rest in part on the name. Brand loyalty in general is central to marketing, although ultimately, in the words of Rotfeld [62], ‘so little is known about how or why advertising works’.

## 4. Verbal and Visual Suggestivity

In the space between direct denotation, or assertion, and suggestivity, lies a further, ubiquitous type of meaning: implication. While calling a drug Allegra asserts nothing and implies nothing about its use or usefulness, and merely seems to suggest something positive, calling a drug Cureflu would imply that it cures the flu. Languages employ an array of tactics for saying something by implication [63,64]. Some cultures, particularly non-Western, make a virtue of indirectness in serious communications where face might be threatened [5,65]. How to draw the line between implication and mere suggestivity is a long-standing problem for linguistics and psychology but a boon to advertisers, and a problem for the FDA. While ‘drug speech’, be it by a brand-name, such as Cureflu, or promotional text, must not claim to cure, even by implication, US regulations allow commercials to *suggest* a beneficial or easy medication experience by verbal, aural, or visual association. The suggestive power of imagery and sound has been well recognized and analyzed in both the semiotic and the marketing literature [66,67].

The FDA has belatedly come round to the view of the AMA’s Council on Ethical and Judicial Affairs [68]:

Many broadcast advertisements are misleading, using imagery to suggest effectiveness far beyond what clinical evidence suggests…

And has recently begun rejecting suggestive visuals for distracting from the risk message, but still leaves ample linguistic and discursive opportunity to influence the medication experience or at least to put the physician under pressure to prescribe the advertised drug [69].

## 5. Strategizing the Power of the Message

Communication offers many ways of strategizing the power of a message, some covert (as with suggestivities) and some more overt. The sender commands a slew of variables to achieve prominence for certain elements of the message, such as the amount of space or time allotted, formatting, and positioning (factor #6: message: organization). The net effect may be to increase attention and persuasion (factor #13: attitude). Importantly for communication ethics, such elements of communication often allow the sender to abjure responsibility for the effects.

Weighting is a further controversial feature of drug advertising to the consumer. Despite the FDA stipulation [68] that broadcast ads maintain ‘fair balance’ between risks and benefits, just the major risks of a medication need to be stated on a broadcast ad. A content analysis [70] of a 2004 sample of TV ads found them to be full of positive appeals—albeit while spelling out the sufferer’s distress—while the risks to the patient were stated briefly and discreetly. A fresh sample in 2016 of 61 TV ads [71] found them still dominated by positive emotional appeals while educative information about the condition (prevalence, mechanisms, and so on) had actually been quietly rolled back. What the actual effects of ‘fair balance’ are in terms of attention, comprehension, recall, or behavior change may be debated, and reported patient satisfaction with such ads may also merit consideration [72], but in communication terms, fair balance is far from being achieved.

## 6. Participant Roles and Relationships, Activities and Actions, Attitudes, and Settings

The roles and relationships of participants in a communication (#2), the activity type and the verbal actions being performed (#4), the end goals (#3), and the setting (#12) are all central to discourse. The effect is often a loop, both affecting and reflecting the proceedings.

Pharmacist–patient communication has not attracted the study it deserves [73]. There is a need to explore the range of pharmacy settings, activity types, genres of discourse [74], and their goals, as well as communicative norms (such as each participant’s allowable contributions), styles, and the tacit inferential schemas on which smooth interaction depends.

Counselling is an activity with a distinctive form of discourse [75] and interactional challenges. Discourse analysis of healthcare counselling by pharmacists and nurses [76,77,78,79] has struggled with such issues as whether there is a clear difference between information and personal advice (‘if I were you’) in the mind of patients and practitioners; whether a professional’s air of expertise effectively bars mutual dialogue; and whether a non-response signals non-uptake. In any event, a key goal of pharmacy counselling, be it information or friendly advice, is safety.

Mixed roles can pose problems for both the pharmacist and the consumer. For example, at in-store pharmacies, dispensing, counselling, and transactions are offered in tandem, sometimes at the same spot by the same person. How are these roles and activities separated or hybridized discursively, in the way the pharmacist and the customer/patient speak, in the store signage, and so on, and how does a pharmacist’s mix of roles affect consumers’ perceptions of the pharmacist as counsellor and their behavior? A critical factor at the in-store pharmacy is the pharmacist’s perceived and actual availability to speak, in person and by phone (#11). Holden and colleagues [80] observed that shoppers sought product information primarily, if at all, from packaging and more rarely (10% of shoppers) from a pharmacist. The ‘embarrassment and ignorance that it was appropriate to seek information from pharmacists’ reported in 1996 [81] may still be widespread. The cumulative effect of patients’ non-use of counselling, resultant dearth of counselling experience for pharmacists, and unconducive store settings may create a communications loop that cannot easily be resolved. A 2003 study of pharmacy visits in New Zealand [82] reported customers’ concern about quality of counselling, empathy, and privacy. Instead, patients may place their confidence in brands or treat OTC medications as safe or “not real” medications [47,83].

A verbal action that poses a particular challenge in the pharmacist’s role as counsellor is the need to rapidly elicit information but without confusing the patients (who may have age- or culture-related difficulties of their own) and without asking leading questions such as ‘Have you noticed any changes since starting this medication?’ (speaking about the benefits and risks of statins) rather than ‘Are you having muscle pain?’ [84].

A different functional setting (#12) for the pharmacist, with a different kind of addressee (older patients) and goal but arguably the same activity, is the home medication review for older patients. The tone (#8) may also be the same. Although such reviews in the home are intended to be rather informal and conversational, the pharmacist’s preoccupation with safety and the task-driven structure of the medication review (introduction, cognitive testing, enumeration of medications) are hardly conducive to ‘conversation’. The ‘protocol-driven discourse involving eliminative questioning that can be technical, perfunctory and impersonal in nature’, which Norris and Rowsell [82] saw as part of a pharmacist’s community of practice, is not easily transformed. Professional communities of practice are characterized by a mutual engagement, a readiness for joint enterprise, and a shared verbal repertoire [85]; such patterns are part of a core professional identity, and are thus highly prized and hard to change.

A corollary of the professional role of a health care provider is a relationship between the health care provider and patient that is primarily professional, asymmetric in terms of authority and respect, deploying technical terminology to a degree, and likely to be non-social. Such relationships, dominating and formal, fit the discursive category +Power +Distant in Roger Brown’s matrix for pronouns and titles of address [86]. Communication between the health care provider and the patient involves a distinctive discourse, quite apart from the provider’s use of technical terminology.

When the provider–patient relationship is one of counselling, as with home medication reviews, significant counselling issues may arise. Discourse analysis of one type of British medication review (and ethnographic interviews with the pharmacists and patients) found little sign of reciprocal discussion [87], and the same emerges from a study of an experimental Australian *Medicines Conversation Guide* [85]. Indeed, the patients in the former study saw domiciliary reviews as a threat to privacy and autonomy. Thus, the setting of the event (in the home) was an irritant to communication. To take one example:

The communicative strategies that pharmacists used to find out about participants’ OTC medicines often left meanings submerged or hidden. This created and perpetuated interactional confusion and misunderstanding, making the process more difficult and the tasks of MR [medication review] unreciprocated.

If non-adherence is seen as an error rather than a choice, older patients may see this as doubting their competence. Here, then, the goal of the professional activity impinges on personal relationships with the patient. As for the pharmacists, both the aforementioned studies found them enthusiastic yet anxious about their training for informal conversations of this kind. Adding a further layer of complexity to the relationship is the issue of the visiting pharmacist’s standing relative to the patient’s doctor and the right to discuss medication choices, a topic traditionally reserved for a doctor.

Turning to doctor–patient communication, there is abundant research on the role of the perceptions and behaviors of both the doctor and the patient and the discourse that reflects and constructs these relationships [88,89,90,91]. Perceptions of a doctor as communicative and empathetic (#8,10)) have been a major predictor of patient satisfaction, while a lack of these skills is a main cause of complaints [92,93]. Important independent variables are activity types and settings, such as the office consult and hospital discharge, private and managed care consults; actions, such as history taking, diagnosis, and prescribing, each with its own distinctive structure, wording, and discursive norms (#11), amounting to distinct genres (#9); the patient’s and the doctor’s demographic and cultural background (#2); and each party’s personality (#2) and communicative style (#10).

An oft-cited and highly critical study [94] of the discursive norms of conversation management in 74 office visits, subsequently replicated [95], found that physicians were silencing the patient in 69% of cases, after just 18 seconds on average, and focusing on the first thing they heard, leaving all else (such as medication issues) to surface haphazardly if at all. Sometimes the doctor’s part in the encounter verges on a monologue. The consult will be brought to a swift end without hearing the patient’s narrative of illness and perceived pathology [96], and in its place, as it were, the doctor will announce a prescription, which may sometimes come across as a mere placebo.

Writing a prescription is a very significant textual act in a consult. It has been seen anthropologically as a kind of written contract, the healer and the person to be healed uniting to undertake a common action. A prescription functions as a legitimation of sickness. It and the medication that it prescribes are also an embodiment for the patient of the doctor’s verdict [97,98]. A prescription is in some sense a final word, and a final flourish that lasts beyond words. After what the patient has heard or missed or just forgotten, this is something concrete that can be borne away or picked up in the form of a medication. As for the doctor (and the patient may sense some of this), to quote Pellegrino [99], ‘the medication indicates the doctor’s concern; it enables him to communicate with patients with lesser education, different values, or different socioeconomic status; it can forestall lengthy discussion of symptoms and their meaning…it is an effective device for parceling the limited time a physician can allot to a patient.…Giving a prescription is also a major source of satisfaction to the physician, since it may be the only way he can ‘do’ something for the patient.’ In communication terms, then, the prescription has been portrayed not just as a genre but as a ‘speech act’ that seeks to reach out, to heal, and to justify one’s role and attitude.

Of course, for a doctor to imagine that a prescription is the only way of ‘doing’ something for the patient is to deny the health benefits of relationship and confidence building, in which casual exchange and even small talk are powerful ingredients.

In the absence of an open or empathetic attitude and tone, regular checking for comprehension has been proposed as a norm. However, one study [100] has reported that physicians treating low-literacy diabetics were checking for comprehension in just 15% of cases. The unwillingness to hear the patient or check for comprehension undercuts key tenets of doctor–patient communication, and not only symbolizes superiority and a lack of empathy but embodies it. The result for the patient will often be frustration and a sense of disempowerment. Medical factors can threaten the entire doctor–patient relationship and communication may break down, as illustrated by Kleinman [101]: “Chronic pain patients are the *bêtes noires* of many health professionals, who come to find them excessively demanding, hostile, and undermining of care. A duet of escalating antagonism ensues, much to the detriment of the protagonists.”

Style of presentation (#10) can further reflect and reinforce a doctor’s detachment from the patient’s needs, particularly where detail and clarity matter. Thus, some studies [102,103,104] have found that a general practitioner prescribing a new medicine often gives the patient an inadequate notion of what they are taking, what it is for, and how to take it, even though this may require very extra little time and the briefest of teach-back sessions.

However, despite so many communicative woes, trust in one’s doctor is generally high, and often translates into positive attitudes (#13) of patients to their medication advice. From 2005 to 2015, doctors were the most trusted source of health information [105]. Here, one could signal the effect of nonverbal communication, as in the satisfaction generated by a doctor’s expressive body language [106].

Widespread changes in medical training, the product of a new paradigm of patient autonomy and shared decision making [107], may augur more willingness among younger doctors to listen and engage, and a more casual, simpler, and everyday style of presentation. There is some awareness of the need to minimize or at least explain medical terms, though the gaps in the vocabulary of many patients extend well beyond medicalese. A promising development is a CDC provisional guide for ‘jargon-free’ language for federal public health-talk [108], which offers simple substitutes for terms like ‘abstinence, acute, adherence, incidence, incubation, optimal, prevalence’.

Such things have been shown to go some way to reassuring patients, uncovering their experiences and expectations of their medications, and working toward shared decision making [109,110,111]. 

Essentially, however, what shared or collaborative decision making actually is, or should be, is not self-evident in terms of discourse: Does ‘shared’ mean ‘equally shared’, and how would this be assessed? And, as mentioned earlier, sensitive discussions often involve negotiation, strategic ambiguity, and even plausible denial. Future medical communication research could benefit from these more nuanced notions of negotiated and conflicted meaning using qualitative discourse analysis [4].

A different consult setting, the private practice, with different rules (e.g., cost, duration), sometimes allows the patient, if so minded, to play an active part in discussion of the diagnosis and treatment. Unlike public clinics, which have been the setting of most doctor–patient studies, private practice often affords longer consults and long-term relationships, giving patients more control of the agenda. Ainsworth-Vaughn’s [53] ethnolinguistic profile of oncological encounters uncovers several simple spontaneous tactics by which patients claim a share of discursive power without threatening the doctor’s face. Deferential disagreement can be signaled by back-channels (*hmm*) and hesitation. A question can be posed discreetly, without subject-verb inversion or explicit intonation; indeed, Ainsworth-Vaughn found that her patients were managing to ask one-third of all the questions in a consultation. Displaced voice is an even stronger way of challenging a diagnosis and proposed treatment: ‘My sister says…’ when she means herself. Telling one’s story or own perspective on events may allow one to define oneself [112] and mitigate painful treatment: ‘Suffering is produced and alleviated primarily by the meaning one attaches to one’s experience’. Further research in the private practice setting is a desideratum.

A provider–patient setting where two-way accurate information-sharing is a pressing need, indeed a requirement for accreditation [113], is hospital admission and discharge. The hospital experience is likely to be unfamiliar, bewildering, even alarming, with different mutual expectations and relationships. Discursive difficulties abound [114], due in part to the lack of a standard for training or patient comprehension assessment [115]. Thus, Dr. Terry Davis describes the incomprehension she herself experienced on discharge after mitral valve surgery, even though she is a professor of medicine [116]. Her physician was unaware that she was going too fast with unfamiliar routine discharge instructions, and Davis was too overwhelmed and embarrassed to ask all that needed to be asked. It has been proposed that ‘discharge’ be treated as an unfolding event, calling for medication counseling by pharmacists [117].

Taking an extreme but all-too-familiar setting, severe communication problems confront the emergency department. The ED environment is often unpredictable, close to chaotic, staff often stretched, and patients stressed, unfamiliar with the clinician, and sometimes never having been to a doctor. Thus, discharge sheets are a critical part of ED treatment. However, Williams and colleagues [118] and Hayes [119] found 45–50% of ED patients were unable to comprehend a common ED discharge sheet, due in part to the large number of low-literate patients in ED. Yet one study found that only 16% of the providers at a teaching hospital ED inquired if patients had questions about the discharge material, and none asked if patients understood their diagnosis or discharge plan [120].

In this section, we illustrated a number of communication factors, in particular: type of activity (e.g., counselling, eliciting information, conversing, prescribing), role (e.g., patient, ED doctor, GP in private practice, pharmacist, mixed roles), social relationship (e.g., patient-pharmacist, lay-expert), setting (e.g., home, in-store pharmacy, admissions, ED), norms of communication (e.g., conversation management), tone (e.g., formal, casual), and attitude to message (e.g., trust, empathy, detachment).

## 7. Traits and Capabilities

The individual and demographic traits (notably gender) of health care professionals have attracted some attention. The influx of women into the medical profession promises to make a major impact on communication in the doctor–patient relationship. More women than men are now enrolled at US medical schools [121]. There is sociolinguistic evidence [122,123] that American women tend to communicate to other women more personally, and generally, less assertively. For example, a woman doctor may be less inclined to change topic unilaterally and may spend longer with a patient [53,124]. There is also some evidence that female patients are more comfortable with a female physician [125,126,127,128]. 

The demographics, individual traits and knowledge, and cognitive and physical capabilities (#2) of the patient impact health communication in many ways. However good the healthcare system and the medication messages, patients may have their own constraints: poverty, advanced age, hearing loss, poor English, medical preconceptions, personal or cultural reserve, fear of ‘wasting the doctor’s time’, and much more. Professional training and health literacy intervention can only go so far.

Some sense of the public’s understanding of formal information about their medications was provided by the broad National Assessments of Adult Literacy (NAAL) survey of 2003 [129]. Much faulted [130] and not re-run since then, it reported that just 12% of the adult US population had ‘proficient’ health literacy, while over one-third had no more than what it defined as ‘basic’ health literacy, insufficient for understanding common medication instructions. Men scored slightly lower than women; those aged 65+ appreciably lower than those aged 25–39; and Blacks and particularly Hispanics raised in Spanish markedly lower than Whites.

Davis and colleagues [116] provided some stark examples of the scale and severity of the problem for America: Of the ‘basic literate’ in their study, only 52% understood ‘*take with*) *plenty of water*’, and just 13% understood the terms ‘*refrigerate*, *shake well*, *discard*’. They may be relying on common sense and a little help from friends, but often they will not think they need help when they do.

Health literacy has preoccupied many health planners, as at the IOM workshop of 2009 [131], and there is greater disagreement than ever about what to measure and how in practice to measure it [132]. Like so many snappy notions, ‘health literacy’ has been deployed to serve the interests of multiple stakeholders. Patients receive formal and informal information, in words and numbers, in a variety of settings, to various ends. Their grasp of texts and numbers is often measured; their speaking and listening less so. Some common measures of textual difficulty have been much criticized. Age and culture compound the problems.

This is hardly where the problems end: The medication information system is also part of the health literacy problem (see Section 10). Even well-educated highly literate people may have trouble with a medical form or doctor’s instructions for a drug or procedure, and if they are older (as so often they are), even the risk labeling on an OTC analgesic may not necessarily be effective [80].

Older people, particularly the very old, are liable to neglect or mismanage their meds, due inter alia to polypharmacy, costs, living alone, physical frailty, and impaired cognition, vision, and dexterity [133,134,135]. When hospitalized, they may receive little medication education or discharge information in writing (often more popular than verbal advice), and coordination with their pharmacist and physician may often be poor, time is short, and discharges may be rushed. Yet, many older people wish to maintain their independence and to be in control of their medication.

Of the 3.8 billion prescriptions written each year in the US, more than half are taken incorrectly, or not at all [136]. Whether responsibility lies more with the patient or with the health system is moot [137]. So many factors are at work, many involving language and discourse. DHSS Healthy People 2030 has now proposed changing the meaning of ‘health literacy’ to emphasize society’s role in providing the information: “Health literacy occurs when a society provides accurate health information and services that people can easily find, understand, and use to inform their decisions and actions” [138,139]. That should be ‘the system’, not ‘society’; family, friends and fellow patients can hardly be held responsible.

Circumstances too can cause communicative disruption, sometimes quite predictably. Thus, in pediatrics, medication errors are generally a result of parents’ or carers’ confusion—the crying child in the middle of the night and the dosage is not clear. Altogether, the psychology of whether someone is a habit follower or a deliberator [8] may predict much more about the effectiveness of the message.

## 8. Sociocultural Effects

The extensive research regarding sociocultural health practices and discourse in the USA [140,141,142] has noted troubling medication miscommunications and outcomes. The American health system, while increasingly committed to cultural competence or sensitivity [140], both domestically and in global health efforts, has been slow to introduce it into the medical school curriculum [65] and is poorly attuned to cultural differences involving medication, for example, with respect to informed consent [143].

However, long-standing interest in traditional Chinese medicine has generated proposals to bridge the clinical gulf between it and Western biomedicine. Kleinman et al. [101] argued that clinicians be trained to elicit the patient’s model of cure while also advocating a biomedical model. In one case study, ”The [Chinese] patient responded to a course of antidepressant medication [at a Massachusetts hospital] with complete remission of all symptoms. He thanked the psychiatrist for his help, but confided that […] perhaps the combination of both traditional Chinese and Western drugs had been responsible for his cure.” The two systems and philosophies of medication can exist side-by-side: In Taiwan, Kleinman and Sung [144] found that ‘Patients expected Western-style doctors to provide injections, but not to spend much time in explanations and in answering their questions. Chinese-style doctors are expected to prescribe herbs and to limit their remarks to discussing symptoms and diet. Unlike Western-style doctors, however, they are expected to respond to questions.’

Some significantly different philosophies (cultures, if you like) of medicine and cure are to be found in Western complementary and alternative medicine (CAM). A large turn to complementary and alternative medicine (CAM) in the US since the 1980s–1990s has attracted substantial research, in part reflecting a new respect for cultural diversity. This turn often involves counter-ideologies and quasi-spiritual values; the approach may variously be presented as complementary or alternative. At times, CAM may bring tensions to the doctor–patient relationship, particularly if patients speak of ‘self-healing’ or reject elements of mainstream medicine deemed essential for public health.

The CAM vocabulary of medication and cure is frequently different or refitted. It sometimes seeks to convey [145] ‘positivity and self-responsibility and quasi-metaphysical notions of self-actualization and self-healing’ and also ‘the need for control; retaining a sense of power within disease and treatment processes; and, seeking inner peace and/or relaxation.’ Metaphors are crucial to the practitioner’s art and to the patient’s understanding [146]. In traditional Chinese medicine, balance is the central metaphor [147]—two pivoted weights, mapping onto yin and yang, and balancing ‘hot/cold, soft/hard, dark/bright’ etc. Cancer treatment, for instance, will be spoken of as a quest for balance and harmony, rather than as battle and struggle with an intruder fought with ‘courage’, the body is cast as a quasi-spiritual system of energies rather than a medicated machine, and cure is a release of energies rather than a mechanical repair.

Classical homeopathy [148,149] fosters a comparable medication relationship and ‘norm of communication’: consults are lengthy, and patients are encouraged to speak at length about their background and symptoms. Homeopathic terminology and philosophy of cure is quite distinct: The ‘remedies’ ‘suggested’ by a homeopath (‘prescribing’ in the US is legally restricted to doctors) may be for acute or constitutional conditions. Patients diagnosed as needing constitutional remedies are often characterized as ‘being’ that substance, e.g., ‘He’s a sulphur, she’s a pulsatilla child’. Adverse effects are not deemed to be side effects but rather ‘aggravations’, in line with the homeopathic philosophy of cure.

## 9. Conversations, Stories, and Self-Expression

Among the genres (#9) of medication discourse we have noted are the consult, the prescription, and the drug commercial. Informal conversations with family, friends, spiritual figures, and fellow ‘sufferers’ about one’s health and one’s meds can strongly influence one’s treatment decisions, and reflect one’s decisions and experiences, the lived experience within the social world [150]. In such conversations, narratives will likely be central and powerful [151,152,153], often producing mutual and co-constructed storytelling [154]. And as Susan Wells [155] has observed, narratives work against expert taxonomies: ‘systems of abstract nouns are not easily incorporated into stories.’ They also allow for multiple voices and alternative or even ambivalent points of view.

A conversational story about one’s own or one’s family’s meds will sometimes fit into a traditional rhetoric and a genre (often culture-dependent), such as ‘troubles telling’ [156], ‘talking about the kids’, ‘dispensing advice’, involving an appropriate context [157], formulaic expressions, and typical discourse ingredients [158]: opening, making points, engineering credibility, shock, suspense etc., achieving a primary goal (e.g., recommending a med or seeking advice), closing. Physical conversation may involve different ‘communal voices’ [159]: an ‘I’ narrator speaking for the group; a ‘we’ narrator; a ‘sequential’ voice in which individuals narrate in turn. Virtual or digital conversation produces other options. 

And often forgotten is the conversation with oneself or the soliloquy, in a multiplicity of forms and genres. Speaking with oneself is for some people and some cultures a valued form of speech. Illness and medication may be the subject of a diary or journal (written or recorded), and a host of literary forms, casting narrative, maxims, lyric, lament, and so on in the form of song, poetry, and prose [112]. There are also the ephemeralia that do not get written down or recorded: ‘talking to yourself, ‘berating yourself’, ‘pitying yourself’, ‘freaking out’, or however we may label them. The medication itself may be a dramatis persona, as when a person asks, ‘Do I control my medicines or do they control me?’

The way people speak about their medications—articulating their knowledge, reasoning, and feelings—has been relatively little studied, though the patient’s perspective in non-adherence to medication has been a growing focus of research. Two other foci have been illness identity and cultural differences.

Feelings and reasoning about medications can run the gamut from positive to negative, closeness to rejection, pride to perceived stigma. How this can be communicated can be illustrated from various parts of a communications model.

We proposed above that cancer patients might be building an illness identity [160] with the help of positive sound-symbolic suggestivities of drug names. Illness identity can be a site of conflict, as in the study of asthmatics by Adams and colleagues [161]: deniers as well as accepters took their medications, but the former prided themselves on their autonomy, dismissing their condition as intermittent, ‘not proper asthma’, ‘chest trouble’, or the like, while the latter accepted the social identity of ‘asthmatic’, e.g., ‘Asthma isn’t an illness. If it is controlled, it becomes a condition that need not affect one’s life greatly’. The patients had ‘constructed their illness and its relationship to their medication’. Choice of words, in a lay setting, with varying ‘audiences’, about both their condition and their medication management was critical to the strategies of deniers and accepters alike: Deniers were refusing their prophylactic medication, expressing graphic fear of steroids or ‘that sort of stuff’. They portrayed their reliever medication as suited to their purported acute ‘bad chest’ (‘Ventolin is used by asthmatics but it’s good for bad chests too’), as merely ‘opening or clearing the tubes’, as less serious than taking tablets, and as not necessary for days on end, hence not creating a feared dependency or any private suspicion that they were indeed asthmatic. This was all a message to themselves. However, there was also talk of ‘embarrassment’ and ‘discretion’ as they needed to message (tacitly) to significant others that they were quite normal. Accepters, on the other hand, saw no stigma in their inhalers, presenting them as just like a pill, e.g., ‘Both contain medicine. There’s no difference’. They accepted the prophylactic as a routine; true, it was a steroid but this was legit and ‘I need to breathe properly, so what can you do?’ And as if to portray the medicine as less ‘medicinal’: ‘I rarely go to the doctors. I just get repeat prescriptions… He knew I was in control’, suggesting that patients may have a complex notion of what counts as a prescription drug. The burgeoning literature on medicational ‘troubles sharing’ on the digital media will add substantially to this relatively small field of study.

The proposed distinction [37] between medicalization, pharmaceuticalization, and psychologization, for example, in the treatment of insomnia and sleep disorders, has a place in patients’ talk (adumbrated earlier) about their ‘illnesses’, ‘aches and pains’, and so on, and what they’re ‘taking’ for them, what they’re ‘on’, etc. There is also, of course, a discourse and language of healthicization, involving exercise, diet, hygiene, and supplements. A communication model will require setting these in a mutual context. Thus, part of the meaning of supplements may derive from portraying them as ‘natural’ or ‘safer’ or even ‘cheaper’. Patients’ descriptions to family or fellow sufferers of the effect of non-medical substances will likely differ sharply from when (or if) they speak of them to their doctor or pharmacist, be it the vocabulary they will use or indeed the display of deference, diffidence, or uncertainty.

Self-expression is not necessarily about personal experience or expectation. If it is the nature of creative expression, indeed all self-expression, to feign or embroider or fasten onto the words of others, how much more so when contemplating treatment and medication. And rather than soliloquize, some may publish or go public, such as the narrative poet Sharon Olds. Here is an extract from The Procedure [162]:

[…] I watched the tray, where he opened packets
with a sterile flourish—scalpel, scissors,
needle, thread like a wild, alive-ish
thing. *How deep do you go, an inch?*
He laughed with amusement. *No! Then we’d be*
*in your spine! I love Novacaine,*
I said, wanting to converse like an equal,
and told him how nylon was named, after
New York and London, and he told me Nystatin
had been named after NY State. Then he carved a little
round of skin out of my back […]

A whole canon and a popular culture is concerned with sickness and medication. The representation of psychiatric medications, starting in the 1950s, has recently begun to attract scholarly attention. The poet Robert Lowell, gradually succumbing to mental illness [163], attached an enduring label to a generation (“Memories of West Street and Lepke”):

These are the tranquillized fifties and I am forty.

But would later find himself an outcast, surviving by tranquilizers he hardly dare name:

Tamed by Miltown, we lie on Mother’s bed (“Man and Wife”)

I am a thorazined fixture/in the immovable square-cushioned chairs (“Home”)

The general depiction in literature and film of mental conditions and their treatment with psychotropic medications is the subject of a recent book by Roger Bennett [164]. Anthony Burgess’s novel *A Clockwork Orange* (1962) has been explored [165] for its counterpoint between the new psychotropic treatment of deviants and the countercultural adoption of psychotropic drugs for consciousness-raising, with each fictional drug functionally and pharmacodynamically pointing to real-world analogues. Words give this counterculture its edge: The gang of ‘Droogs’, empowered by their argot Nadsat (a riposte to Newspeak), construct an identity from fashion, drugs, and slang: Milk plus knives (subverting the slogan ‘Drinka Pinta Milka Day’), they take *vellocet* (cf. velocity, speed), *synthemesc* (cf. mescaline, synthetic), and *drencrom* (cf. adrenochrome).

Forty years on, HBO’s series *The Sopranos* was iconic of “a newly emergent genre, which I call the psychopharmacological thriller” [166], in which “new narrative techniques are being developed to probe the inner neurochemistry of the human brain, how medications alter that neurochemistry, and the wide-ranging questions raised by this brave new proliferation of psychopharmacology [Prozac, in particular] in everyday life.”

Some may turn to God, in supplication, confession, guilt, and hope. The role that these play in medication and healing has been affirmed, debated, and also queried [167,168]. Vouchsafed to a health professional or healer, they may offer special insight into the patient’s condition and inner world. And parallel to all this expression of the self is another creative literature, portraying (ostensibly) the medication experience of others, but oftentimes subtly autobiographical [169,170]. An annotated database of literature, arts, and medicine (LitMed) is curated by the NYU Langone Medical Center.

## 10. When the Information Source Is Far from Obvious: Leaflets and Labels

We have portrayed knowledge about use of a medication and its benefits and risks as coming from a variety of sources (expert, lay, personally, remotely, known or unknown to the user) and in a variety of settings, genres, and mediums. The impacts on the communicative outcome (such as involvement, trust, comprehension, recall, action) are complex.

Formal written medication information for the patient is one group of genres with a distinctive communicative profile: The information source is anything but obvious [8]: FDA-approved prescribing information, in part, but usually with content from manufacturers, pharmacy chains, or undeclared information vendors, and sometimes regulated by state boards of pharmacy. The ostensible addressee is sometimes lay but at other times a combination of lay and expert. The information reaches the American end-user in a variety of settings, genres, and mediums, notably the label, MedGuides, pharmacy leaflets, package inserts, printed ads, and on-line as website content (brand-sponsored or other). The label, given its function and position, is ostensibly intended to be basic and user-friendly. The others too might be expected to be user-friendly, particularly in the frame of an attractive print ad or website.

This, however, is not the case. The content, language, and format of all these genres are frequently impenetrable, even for highly literate Americans. MedGuides, for example, were found to be ‘of little value to patients’ [171]. So, it may not matter, ironically, whether these guides are even reaching the patients (Shrank and colleagues [172] raised doubts). Regarding leaflets, less than a third of patients have been found to attend to the leaflet stapled to their prescription bag and frequently discarded it [116]. One obvious reason for this attitude is that the information *looks* so daunting with its technicalities, fine print, and sheer length. To achieve clarity, brevity, and comprehensiveness in one document would seem to be a mirage.

Instead of the leaflet or insert, many will rely for guidance on the labelling, especially with OTC medicines. However, the labelling has been identified as a major cause of errors [173]. Even if your eyes (and the lighting) are good enough to decipher the print, literacy is no safeguard: One study [116] found that over one-third of patients with adequate literacy, given five pill labels, failed to understand at least one of them. While limited literacy is associated with misunderstanding, the instructions themselves were “awkwardly phrased, vague, and unnecessarily difficult” [173]. Readability scores and simplicity are no guarantee: “take with food” is written at a first-grade level, yet 16% of 395 patients ranging from marginal to adequate literacy did not understand it [173]. Only 59% understood the intention of “Medication should be taken with plenty of water”, which fails to define *plenty*. Just 8% understood the unfamiliar complex instruction “Do not take dairy products, antacids, or iron preparations within one hour of this medication”. Disastrously inadequate labelling of OTC medicines containing acetaminophen was only rectified in 2013 [174]. Some errors could easily be avoided [175], e.g., by changing the standard chain pharmacy instruction TAKE TWO TABLETS TWICE DAILY (a phrase that doubtless sounded crisp and clear to its author, despite the ambiguity in the scope of the word ‘two’). The corrected message—‘Take 2 pills in the morning and 2 pills at bedtime’—may not score highly on readability tests, but it is a whole lot clearer. A token of the growing concern about labelling was the Institute of Medicine’s 2008 roundtable workshop entitled Standardizing Medication Labels [176].

The notion of a ‘label’ as a genre draws on a long rhetorical tradition of recapitulation and summarization [177], a verbal (and formatting) skill that is as crucial today as ever. Although a communicative style of clarity, concision, and accuracy is highly valued in the Western paradigm of scientific-technical discourse [178], many of the challenges and quandaries in creating effective labels and summaries remain the same, whatever the content [179].

One chronic issue is standardization. A label would seem to invite uniformity, for mass use and functionality. This is one of its chief communicative goals (#3). The medication label, in particular, is there to inform and direct, not to please; users need to be able to quickly locate and grasp the information. And an aid to this is that the label look and sound simple and familiar, a meaning at a higher semiotic rather than verbal level.

However, the realities of the US medication label are otherwise. An American College of Physicians white paper on drug labelling [180] found that national pharmacy chains had 31 different label styles, some of them complex and unclear. Davis [181] reported a huge variability in the language of dosing instructions and in icons and auxiliary label colors. As for pouring a child the right dose, a challenge for distracted parents at the best of times, a study of 200 top-selling pediatric oral liquid OTC medications [182] found inconsistencies between dosing directions and measuring devices (missing or superfluous markings) in 98.6% of cases, as well as a mix of teaspoons, tablespoons, and milliliters. Equally serious is the tiny print on that Tylenol bottle or packet, from which you should know how many tablets you can give a child. Factoring in the entire communicative situation, such as the likelihood of dosing the medicine in the semi-dark in a frantic state of mind, the probability of mis-dosing will be high (FDA guidance was belatedly issued [183]). The COVID-19 outbreak, limiting access to pharmacies and clinics, has made the label even more critical [184].

Standardization has become a popular if dubious goal. The FDA has called to harmonize package insert designs and to conduct continuous testing [185], and has endorsed a uniform Drug Facts Label for OTC medicines. The goal was a simple table format in plain English, akin to the Nutrition Facts Box found on processed food packaging [179], a marriage of familiarity with simplicity. Similarly, for prescription drugs, a Fact Box label was proposed in 2012 by Woloshin and Schwartz [186], using an appealing table format for quick access. There is some cognitive evidence [187] of the all-round superiority of such tables over Q-and-A’s or bulleted highlights.

However, users’ experience of medication labels and leaflets is still far from satisfactory. To harmonize is only one step in a daunting quest. The FDA has conceded [188] that no standardized text can speak to all users. Even the preferred length is an issue. Effective simple writing is an art as well as a science, and a particular challenge for technical writers reared on STEM. Even in the EU and Australia, well ahead of the US in leaflet design and testing, industry compliance with the new writing regulations has been patchy [189,190,191,192]. A recent multi-country meta-study of patient preferences [193] revealed dissatisfaction and a wish for medication leaflets to be customized, clarifying how, how much, and how swiftly the drug would help. The authors conclude with a possible resolution: ‘At the very least two versions should be available; a summary leaflet plus access to more comprehensive information if desired’. Another mixed verdict comes from a recent randomized trial of fact boxes [178]: “Participants regularly want more detail on presented evidence, but it remains unclear how to balance clarity and brevity with comprehensiveness.” What the authors should have said is that all of these are variously necessary, depending on the users and their varying needs. There is, of course, a plethora of other information on the Internet; but, so far, its potential for customizing medical information has not been tapped. Used judiciously, the Internet could revolutionize medication information; used unwisely, it can make things much worse.

## 11. Discursive Issues in Risk Communication

One particular function of patient information, to communicate risk, should be addressed separately, as bringing some difficult discursive and linguistic issues regarding medications to the fore. Risk communication in general [194] is a field with many distinctive applications to health discourse [195], and this can be applied to risk conveyed by a doctor or pharmacist.

Discourse analysis of the first generation of British package inserts (predating American inserts) found an intriguing discursive mix [196]: In format, adverse effects were listed transparently, yet packed discretely in running text with few graphic highlights. Is this an acceptable way to square humanitarian and commercial interests? In terms of the tone and ostensible goal, the package inserts wavered between warning and reassurance. Stylistically, they cycled between science and simplicity. The anonymous author(s) may have been torn between two conceptions of risk communication, or they may have chosen to blend the two, or more likely the package inserts were the work of multiple hands. Linguistically, there was an unfortunate indeterminacy about the use of ‘may’ or such descriptors as ‘common, rare’, and the cautions and instructions themselves were sometimes vague or ambivalent. In fact, vagueness is a common feature of the medication risk information literature. Change is unlikely, given industry pressure to template risk information combined with a shortage of evidence about what works best and when.

The relative merits of quantitative and qualitative descriptors of risk is problematic. One study has argued that quantitative descriptors of risk are necessary in weighing risk and benefit because qualitative descriptions are too ambiguous [197], though it can be objected that less numerate women in this study could not accurately determine the benefits of screening from the numbers provided [198]. A questionnaire-based study [199] of risk-minimizing language in ads, such as ‘mild, short-lived’ and the verb ‘may’, found it ‘vague’ and ‘misleading’. Is it possible to ‘regulate’ the problem away? The *Rules Governing Medicinal Products in the European Union* [200] seek to do just that: Disallowing language like “well tolerated” and “normally rare”, they require the following quantifiers of risk (or the equivalent in other languages): Very common (≥1/10); common (≥1/100 to <1/10); uncommon (≥1/1000 to <1/100); rare (≥1/10,000 to <1/1000); and very rare (<1/10,000). However, Cox’s stratified national sample hugely amplified these values [201]. Only unremitting effort can change language habits. The FDA has not tried, and probably should not.

Video commercials pose a special problem: time constraints, linearity, and suboptimal attentiveness can render risk information less effective; and it is limited to major risks. Additionally, despite the rule that risk and benefit be equally prominent, the audio or visual risk text does not easily compete with the visuals and audio content designed to promote the product [72,202].

How physicians conduct informed discussion about the risks versus benefits of medication will be influenced by multiple factors. The patient’s knowledge and involvement can play a part. Thus, some patients may be much more ‘media-informed’ and communicatively active [203]. Discussing risks and benefits over more than one encounter may also be a useful part of the management of the message [204].

Top of Form.

Bottom of Form.

## 12. The Internet

The Internet and social media have rewritten the communication rule-book, and opened a new and richly researched era in health communication. Many websites are a difficult fit for the standard model of communication presented earlier, in particular source and addressee, genre, medium, and communicative function. Web sites are probably like no other genre of text that we are familiar with. Space only permits some brief considerations. Many of the things this paper has highlighted are there, reframed or reinvented. Many online genres and types of interaction are quite new, such as social networks [205] or www.patientslikeme.com (accessed date 15 December 2020) [8,206], a website about others with the same medication or condition on which hundreds of thousands of members have shared personal health data and experiences. The age-old tug between listening to authority or to ‘people’ has now been magnified with the social media’s bandwagon effect [207].

The Internet holds out possibilities for tailored information for different demographics, different language capabilities, and even for individuals. Ethnicity or income do not currently seem to create disparity in Internet use except among the aged [208,209]. However, many websites are a navigational maze, which upsets normal reading and skimming and makes attribution of authority harder than ever [8,210]. Users generally scan rather than read; they read no more than 25% of the text on a visit to a page; and they only notice the first few words of a link before deciding to visit it. Focused search and study is possible, but there is much about many websites that disarms one’s critical faculties. Are ‘official’ brand sites a sort of professional-to-layperson communication? Maybe they are intended to be, but authority is backgrounded and often vague, with little reference to experts. Whose ‘voice’ does the user think this is, industry or the medical profession? A second distinction that often gets blurred is product information vs. health information. For example (at one blockbuster site), the left-hand link bar offered a clear three-way distinction: product ~ condition ~ wellness. Yet, at the first and second tier navigation bars, this distinction was muddled, while the dominant graphic itself (perhaps the first thing users look at) took the form of a river of random links, some about the product, some about the condition, and some about healthy living. A third blurring is between education and promotion, and it is not uncommon for ostensibly educational content to be couched in ad-speak (e.g., chopped sentences, repeated phrases, second person address, emotive phrasing), alternating with measured expository prose. Future study should apply general website usability research [211] to these kinds of issues.

The FDA has not totally risen to this challenge; currently, the same rules that applied to broadcast advertising are being applied to Internet and social media channels. The FDA has, however, ruled in the name of fair balance that risks and benefits must be listed on the same web page; and that even space-limited platforms must state risks and link to fuller risk information [212]. Meanwhile, however, as the Internet and social media become ever more pervasive, so too do the insidious blends of on-line information and promotion, often not marked as ‘infomercial’, often in a bewildering mix of text, image, and video, or in a short tweet or YouTube clip.

## 13. Conclusions

We have taken the notion of a ‘medication experience’ broadly, to include the patient’s expectations and impressions as well as what he or she experiences. There is theoretical value in taking these together, in terms of what people say and hear about their medications and how they variously interpret and absorb it, act upon it, or appear to ignore it.

Communication about medications is central to health and the health system. A clearer understanding of it has the potential to enhance the relationship between patients and providers, decrease cultural and social disparities, combat misuse of medications, and improve health outcomes.

This paper has sought to illustrate the power of a standard model of communication, evolved by linguists, ethnographers, and sociolinguists, to integrate a broad selection of studies in medication communication and highlight the complex interplay between actors, messages, and contextual factors underlying the medication experience, for the benefit of health researchers for whom language-related fields are perhaps unfamiliar.

Our analysis has thrown up many issues and phenomena that merit more investigation, and perhaps cross-disciplinary collaboration. To take a few examples: Can sound symbolism of drug names be shown empirically to influence how they are perceived? What kind of interactions take place in pharmacies and how do pharmacists’ hybrid roles impinge on this? Is patient–doctor talk more ambivalent or negotiable than normally thought? How might demographic factors impinge on vaccination hesitancy? Some issues, such as the blurring of promotion and information on drug brand websites, the conflict between concision and clarity in labelling, or the chronic difficulties in producing usable medical literature, are cause for great concern and call for fundamental rethinking.

Language, as authors and advertisers are forever demonstrating, is unfathomably complex and malleable. Sometimes communication falters, even fails. More often than not, it works. How, exactly, is far from clear, but the ever-growing interest in the place of language in constructing our world holds the promise of much new insight.

## Figures and Tables

**Table 1:** Factors in an ethnographic/social-semiotic communication model.

#	Factor	Example
1	sender	anonymous text, speaker
2	participant characteristics	demographics/culture, personal traits and capabilities, roles, relationship
3	goals	health outcomes, sales
4	action, activity	warning, diagnosing, counselling, selling
5	medium	channel, language
6	message	content, theme, form, organization, thrust
7	register (realm)	medicine, religion, chit-chat
8	tone	modest, open, blunt, authoritative, distant, casual, flippant
9	genre	prose, poetry, leaflet, letter, ad, joke
10	communication style	degree of directness, detail, concision, clarity
11	norms	rules of interaction, accessibility
12	setting	website, pharmacy, clinic
13	attitude to message	attention, involvement, respect, certainty, trust

## Data Availability

Not applicable.
